# Medicinal Soap

**Published:** 1890-05-17

**Authors:** 


### THERAPEUTICS.

### MEDICINAL SOAP.

The British Pharmacopœia contains several kinds of soap, viz., curd soap, made with soda and a purified animal fat, principally stearin. This soap is used in the preparation of several kinds of plasters, suppositories, &c. Next we have hard soap, or "white Castile soap," made with soda and olive oil, and entering into the composition of various pills. Soft soap is composed of potash and olive oil, and used in manufacturing turpentine liniments. Briefly, all soaps are a combination of an alkali and a fatty acid. When soap is dissolved in water, some of the alkali is set free, and uniting with the exudation on the skin, removes it. Owing to their affinity for waters, and their solvent action on nitrogenous tissues, several of the group of alkalies, by abstracting the constituent water, will destroy the skin and under-structures to a considerable depth, but the alkalies used in the manufacture of soap are comparatively feeble in this respect. Often soaps show a bloomlike deposit on the surface, which is the alkali "sweated out," in the language of soap manufacturers. In a late *American Druggist* to hand there is a timely caution with regard to soap. The writer says when the soap contains an excess of alkali the action on the skin will be very powerful, and adds, "None but dermatologists can have an adequate idea of the harmfulness of soaps. . . . But it is in the treatment of skin affections that an uncompromising protest should be entered against soaps, for they invariably make matters worse. . . . Some of the highly-scented soaps in the market are as objectionable as they can be." On the Continent considerable attention has, during during recent years, been paid to medication by means of soaps, and Mr. Stiefel, of Offen-bach-on-the-Main, has brought out a number of medicinal soaps, which are procurable from his agent, Mr. Richards, Holborn Viaduct. In "Medical Reprints" it is stated that, prominent among the varieties, is a sublimate soap containing half per cent. of corrosive, in accordance with the suggestion of Professor Bergmann. This is highly recommended for the treatment of scabies, *pediculi capitis*, &c. In the use of this soap the inconvenience and disagreeableness of ointments is avoided. Simply washing the body, as with any toilet soap, is sufficient. An aromatised sulphur soap, it is said, possesses the advantages of containing sulphur in the finest and purest state, and of giving out no disagreeable odours. Although ordinary common soaps often do injury, there is no doubt that soaps can and should be made and medicated in such a manner as to prove very beneficial.

